# The mitochondrial genome of the wolfberry fruit fly, *Neoceratitis asiatica* (Becker) (Diptera: Tephritidae) and the phylogeny of *Neoceratitis* Hendel genus

**DOI:** 10.1038/s41598-017-16929-7

**Published:** 2017-11-30

**Authors:** Yun Su, Yue Zhang, Shiqian Feng, Jia He, Zihua Zhao, Zhenzhen Bai, Lijun Liu, Rong Zhang, Zhihong Li

**Affiliations:** 1 Department of Entomology, College of Plant Protection, China Agricultural University, Beijing, 100193 China; 2 The Institute of Plant Protection, Ningxia Academy of Agriculture and Forestry Sciences, Yinchuan, 750002 China

## Abstract

*Neoceratitis asiatica* (Becker) (Diptera: Tephritidae) is one of the most important fruit pestsof wolfberry which is a traditional Chinese medicinal herb. We characterized the complete mitochondrial genome of *N*. *asiatica* and described its organization in this study. This mitogenome had a total length of 15,481 bp, consisting of 13 protein-coding genes, 2 rRNA genes, 22 tRNA genes and a non-coding region (A + T-rich control region). The overall base composition of *N*. *asiatica* in descending order was 40.6% A, 8.5% G, 38.4% T and 12.6% C. The phylogenetic relationships shows that *Ceratitis capitata* and *N*. *asiatica* may be sister taxa. This is the first report of the complete mitochondrial genome of a member of the *Neoceratitis* Genus and the complete mitochondrial genome sequence may provide useful information for phylogenetic analysis and studies between the genera *Ceratitis* and *Neoceratitis*.

## Introduction

The genus *Neoceratitis* Hendel is a predominantly afrotropical group with one species in Asia^1^, which partly distribute in Northwest China (Ningxia, Qinghai, Xinjiang and Inner Mongolia), Kazakhstan and Turkmenistan^2^. *Neoceratitis asiatica* (Becker) (Diptera:Tephritidae) is one of the most economically important fruit pests damaged the fruit of the *Lycium turcomanicum* Turcy (Solanaceae)^2^. The majority host plant, wolfberry, is a traditional Chinese medicinal herb and local cash crop^3^. The female adults only lay one egg in an unripe fruit, which exacerbates the destructive power of *N*. *asiatica*. The larvae feed on the wolfberry and develop with the ripening of wolfberry fruit. Once be damaged, the damaged maggot fruits cannot be used as a commodity, so maggot fruits rate can represent the loss rate. Wolfberries damage rate will reach 22–55% if not controlled by using pesticide^4^. In view of the seriousness of the damage to wolfberry, the research on *N*. *asiatica* (Becker) should be increasingly extensive and in-depth. However, the research on the genus *Neoceratitis* Hendel is very limited.

Mitochondrial genomes of insects have been very extensively studied. They have been applied particularly to studies regarding phylogeny and evolution^5–7^. To date there are fifty-seven complete mitogenomes of 23 Tephritidae species in GenBank (Supplementary Table S1).

Currently, studies on the mitochondrial genome of the genus *Neoceratitis* are mainly limited on the species *N*. *cyanescens* by fragments of four mitochondrial genes and one nuclear gene (*COI*, 16*S*, *tRNA* ^*pro*^, *ND6*, *period*)^8–10^, while another important species *N*. *asiatica* (for this study) have not been published yet. Based on the research of *N*. *cyanescens*, we found that the genus *Ceratitis* has a close relationship to the genus *Neoceratitis* ^8–10^, but the phylogenetic status of the two genera cannot be explained very well.

In this study, we reported the first complete mitogenome of *Neoceratitis* species-*N*. *asiatica* and compared the mitogenome data with other tephritid species, aiming to providing more data to study the molecular phylogeny of Ceratitidinain particular.

## Results

### Mitochondrial genome sequencing and assembly

An Illumina library of *N*. *asiatica* was sequenced on a run of Hiseq 2500. After excluding the low quality value reads (lower than Q20), 466,428 read-pairs were generated finally. Through “map to reference” strategy to map all cleaned NGS reads to part of *cox1* gene by Geneious R10.0., 58,875 reads were assembled to get the target sequence. After generating all assembled reads, a consensus sequence length 16,074 bp was generated. Then we manually examined for repeats at the beginning and end of the sequence to form a circle to gain the complete mitochondrial genome sequence of *N*. *asiatica* which was 15,481 bp.

### Mitogenome features

**Figure 1:**
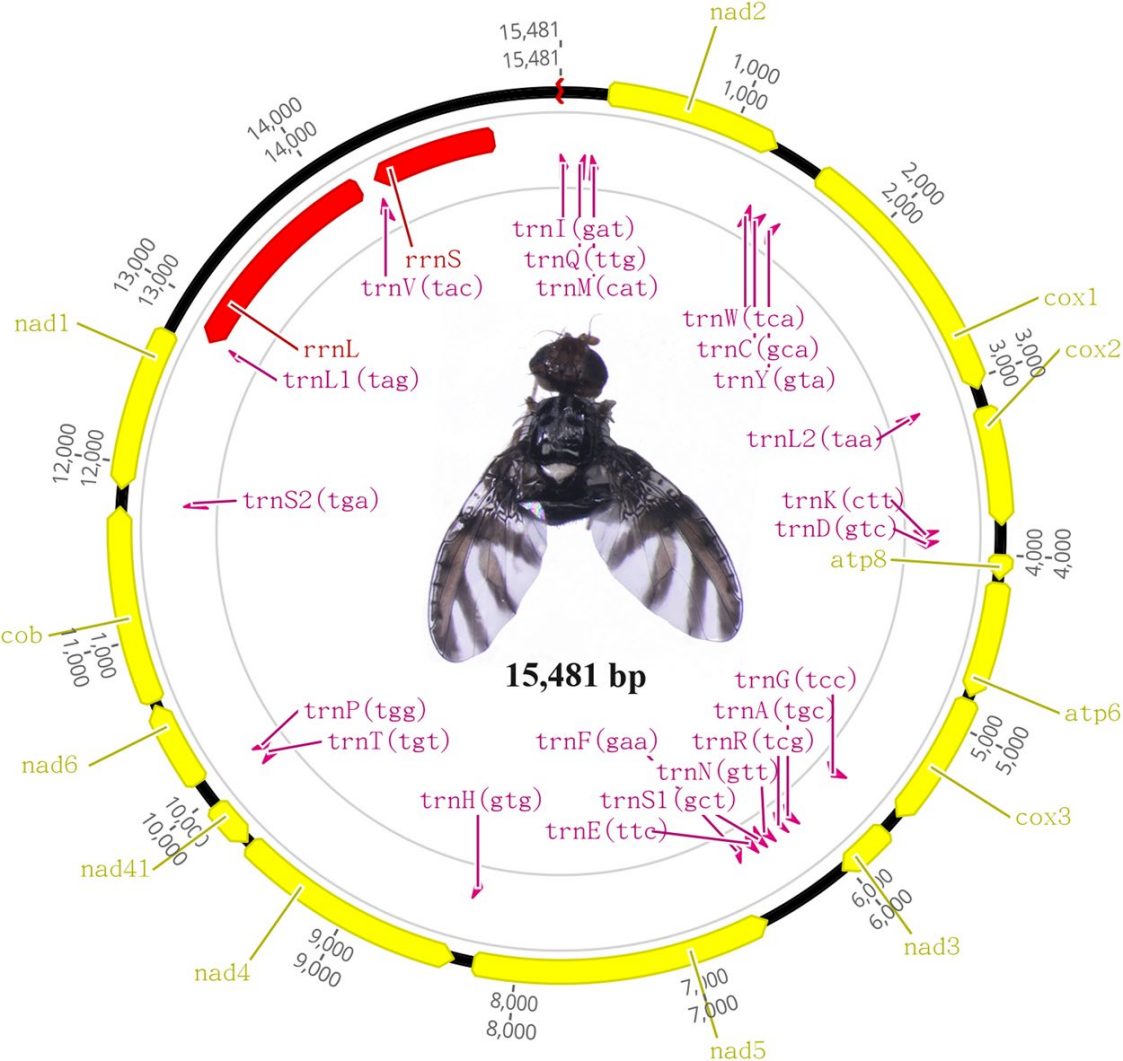
Mitochondrial genome map of *Neoceratitis asiatica*.

The complete mitogenome of *N*. *asiatica* was 15,481 bp in length. The gene content was typical of other ancestral insect mitochondrial genomes (Fig. 1 and Table 1): 13 protein-coding genes (PCGs), 22 transfer RNA (tRNA) genes, two ribosomal RNA (rRNA) genes and a non-coding region (A + T-rich control region). Nine PCGs (*ND2*, *COI*, *COII*, *COIII*, *ATP6*, *ATP8*, *ND3*, *ND6* and *CYTB*), 14 tRNAs (*tRNA* ^*Ile*^, *tRNA* ^*Met*^, *tRNA* ^*Trp*^, *tRNA* ^*Leu*(*UUR*)^, *tRNA* ^*Lys*^, *tRNA* ^*Asp*^, *tRNA* ^*Gly*^, *tRNA* ^*Ala*^, *tRNA* ^*Arg*^, *tRNA* ^*Asn*^, *tRNA* ^*Ser*(*AGN*)^, *tRNA* ^*Glu*^, *tRNA* ^*Thr*^ and *tRNA* ^*Ser*(*UCN*)^) and the control region were located on the major strand (J-strand). Four PCGs (*ND5*, *ND4*, *ND4L* and *ND1*), eight tRNAs (*tRNA* ^*Gln*^, *tRNA* ^*Cys*^, *tRNA* ^*Tyr*^, *tRNA* ^*Phe*^, *tRNA* ^*His*^, *tRNA* ^*Pro*^, *tRNA* ^*Leu*(*CUN*)^ and *tRNA* ^*Val*^) and two rRNAs (*lrRNA* and *srRNA*) were located on the minor strand (N-strand).

**Table 1:** Characteristics of the mitochondrial genome of *Neoceratitis asiatica* (Becker).

Gene	Strand	Location	Size (bp)	Anticodon	Codon		Intergenic Sequence
					Start	Stop	
*tRNA* ^*Ile*^	J	1–68	68	GAT			
*tRNA* ^*Gln*^	N	112–180	69	TTG			43
*tRNA* ^*Met*^	J	200–268	69	CAT			19
*ND2*	J	269–1291	1023		ATT	TAA	0
*tRNA* ^*Trp*^	J	1298–1365	68	TCA			6
*tRNA* ^*Cys*^	N	1358–1427	70	GCA			−8
*tRNA* ^*Tyr*^	N	1482–1548	67	GTA			54
*COI*	J	1547–3082	1536		TCG	TAA	−2
*tRNA* ^*Leu*(*UUR*)^	J	3091–3156	66	TAA			8
*COII*	J	3171–3857	687		ATG	TAA	14
*tRNA* ^*Lys*^	J	3865–3934	70	CTT			7
*tRNA* ^*Asp*^	J	3935–4002	68	GTC			0
*ATP8*	J	4003–4164	162		ATT	TAA	0
*ATP6*	J	4158–4835	678		ATG	TAA	−7
*COIII*	J	4835–5623	789		ATG	TAA	−1
*tRNA* ^*Gly*^	J	5634–5701	68	TCC			10
*ND3*	J	5702–6055	354		ATA	TAA	0
*tRNA* ^*Ala*^	J	6058–6122	65	TGC			2
*tRNA* ^*Arg*^	J	6145–6208	64	TCG			22
*tRNA* ^*Asn*^	J	6250–6317	68	GTT			41
*tRNA* ^*Ser*(*AGN*)^	J	6318–6385	68	GCT			0
*tRNA* ^*Glu*^	J	6386–6453	68	TTC			0
*tRNA* ^*Phe*^	N	6472–6539	68	GAA			18
*ND5*	N	6538–8259	1722		ATT	TAT	−2
*tRNA* ^*His*^	N	8278–8343	66	GTG			18
*ND4*	N	8350–9690	1341		ATG	TAA	6
*ND4L*	N	9690–9980	291		ATG	TAA	−1
*tRNA* ^*Thr*^	J	9983–10046	64	TGT			2
*tRNA* ^*Pro*^	N	10047–10113	67	TGG			0
*ND6*	J	10116–10640	525		ATT	TAA	2
*CYTB*	J	10640–11776	1137		ATG	TAG	−1
*tRNA* ^*Ser*(*UCN*)^	J	11775–11841	67	TGA			−2
*ND1*	N	11857–12796	940		ATA	T-	15
*tRNA* ^*Leu*(*CUN*)^	N	12807–12871	65	TAG			10
*lrRNA*	N	12840–14198	1359				−32
*tRNA* ^*Val*^	N	14224–14295	72	TAC			25
*srRNA*	N	14295–15084	790				−1
A + T rich-region	J	15085–15481	397				0

Spacing sequences in 19 regions ranged from 2 to 54 bp, the longest located between *tRNA* ^*Cys*^ and *tRNA* ^*Tyr*^. The overlapping sequences ranged from 1 to 32 bp in 10 regions, the longest was between *tRNA* ^*Leu*(*CUN*)^ and *lrRNA*.

Contrary to other insect mitogenomes^11^, the nucleotide composition of *N*. *asiatica* was negative AT skews in the control region, while the rest was all AT biased and positive AT skews and negative GC skews in the whole mitochondrial genome, PCGs, rRNAs, tRNAs and the control region (Table 2). The A + T content of the non-coding control region was 88.2%.

**Table 2:** Nucleotide composition of the mitochondrial genome of *Neoceratitis asiatica* (Becker).

Region	A%	C%	G%	T%	A + T%	G + C%	AT skew	GC skew
Whole mtDNA	40.6	12.6	8.5	38.4	79.0	21.1	0.028	−0.194
PCGs	39.9	13.1	9.1	37.9	77.8	22.2	0.026	−0.180
tRNAs	39.9	12.5	9.6	38.0	77.9	22.1	0.024	−0.131
rRNAs	42.7	12.0	6.5	38.8	81.5	18.5	0.048	−0.297
CR	42.1	9.3	2.5	46.1	88.2	11.8	−0.045	−0.576

The commonest start codon was ATG (in 6 PCGs –*COII*, *ATP6*, *COIII*, *ND4*, *ND4L*, *CYTB*), followed by four for ATT (*ND2*, *ATP8*, *ND5* and *ND6*), followed by two for ATA (*ND1* and *ND3*) and one for TCG (*COI*). Ten PCGs (*ND1*, *COI*, *COII*, *ATP8*, *ATP6*, *COIII*, *ND3*, *ND4*, *ND4L* and *ND6*) had TAA stop codon, one PCG (*ND3*) had TAT, one PCG (*CYTB*) had TAG, while *ND1* had incomplete stop codons T.

**Figure 2:**
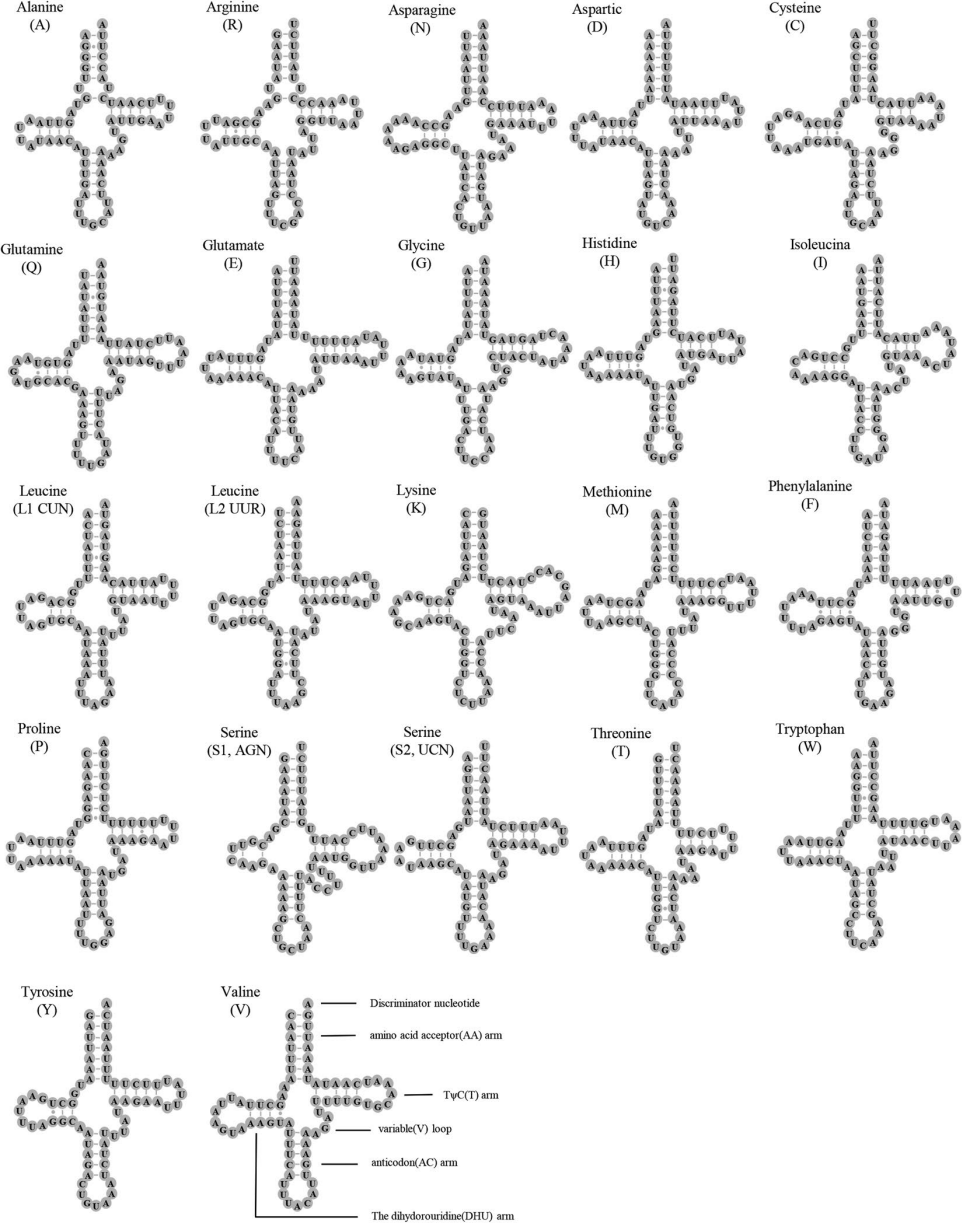
Putative secondary structures of tRNAs found in the mitochondrial genome of *Neoceratitis asiatica*.

The size of 22 tRNAs ranged from 64 bp (*tRNA* ^*Arg*^ and *tRNA* ^*Thr*^) to 72 bp (*tRNA* ^*Pro*^). Most tRNAs could be folded into the cloverleaf structure except for *tRNA* ^*Ser*(*AGN*)^, which lacked the D-loop(Fig. 2). The number of base pairs in the DHU-stem ranged from 3 to 4 (Fig. 2). Most of the TΨ C-stems had 5 base pairs while 7 tRNAs (*tRNA* ^*Ile*^, *tRNA* ^*Lys*^, *tRNA* ^*Arg*^, *tRNA* ^*Ser*(AGN)^, *tRNA* ^*Thr*^, *tRNA* ^*Cys*^, *tR NA* ^*His*^) had 4 bp in the TΨ C-stems. The number of bases in the D-loop and TΨ C-loop was variable.

The two genes encoding the small and the large ribosomal subunits were located between *tRNA* ^*Leu*(*CUN*)^ and *tRNA* ^*Val*^, and between *tRNA* ^*Val*^ and the control region. The *lrRNA* was 1,359 bp long with an A + T content of 82.6%, and the *srRNA* was 790 bp long with an A + T content of 79.5%.

The control region (397 bp) was flanked by *srRNA* and *tRNA* ^*Ile*^ and was highly enriched in AT (88.2%).

### Phylogenetic relationships

Six datasets were used to build phylogenetic trees: 1) PCG123: 13 protein-coding genes (all three codon positions included) with 11,048 nucleotides; 2) PCG123 + rRNA: 13 protein-coding genes and 2 rRNA genes with 12,834 nucleotides. 3) PCG123 + rRNA + tRNA: 13 protein-coding genes, 2 rRNA genes and 22 tRNA genes with 14,186 nucleotides. 4) PCG12: 13 protein-coding genes (first two codon positions included) with 7,342 nucleotides; 5) PCG12 + rRNA: 13 protein-coding genes and 2 rRNA genes with 9,117 nucleotides. 6) PCG12 + rRNA + tRNA: 13 protein-coding genes, 2 rRNA genes and 22 tRNA genes with 10,473 nucleotides.

**Figure 3:**
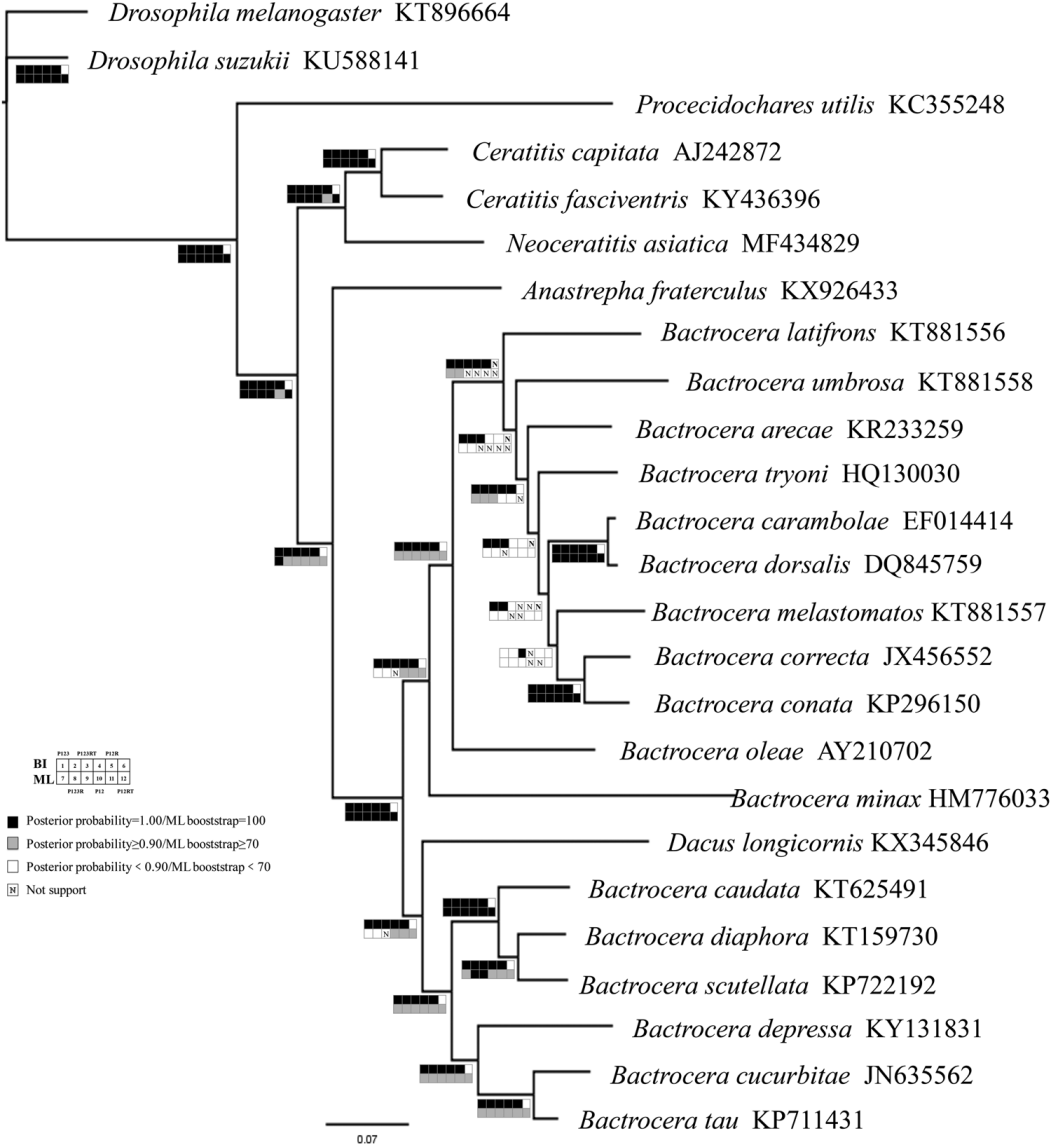
Phylogenetic tree of Tephritidae family based on mitochondrial genomes.

Based on the datasets, the topology structures conducted from Bayesian and ML analyses were very similar (Fig. 3). From our results, the genera *Ceratitis* and *Neoceratitis* are sister groups in the trees with high posterior probabilities (1.0) and ML bootstraps (100).

## Discussion

In this study, we are reporting the first complete mitochondrial genome of *Neoceratitis* species –*N*. *asiatica* (Becker) in Tephritidae. The mitochondrial genome of *N*. *asiatica* is a closed circular molecule of 15,481 bp, which is the shortest one among the other 22 tephritid mitogenomes available with the size ranging from 15,687 bp in *B*. *tau* to 16,253 bp in *D*. *longicornis*. The control region of *N*. *asiatica* mitogenome is 397 bp in length, which is also the shortest one in the other published tephritid mitogenomes with the size ranging from 801 bp in *B*. *tau* to 1,343 bp in *D*. *longicornis* (Supplementary Table S2).

The A + T contents of the whole mitogenome, PCGs, tRNAs, rRNAs and CR in *N*. *asiatica* are 79.0%, 77.8%, 77.9%, 81.5% and 88.2%, well in the range of amongst all reported tephritid mitogenomes, which range from 67.28% (*B*. *minax*) to 80.83% (*P*. *utilis*) in the whole mitogenome, from 64.30% (*B*. *minax*) to 78.90% (*P*. *utilis*) in PCGs, from 72.31% (*B*. *minax*) to 80.61% (*P*. *utilis*) in tRNAs, from 73.71% (*B*. *minax*) to 85.69% (*P*. *utilis*) in rRNAs and from 77.65% (*B*. *minax*) to 91.14% (*C*. *capitata*) in CR (Supplementary Table S2).

The AT skews and GC skews of *N*. *asiatica* in the whole mitogenome, PCGs, tRNAs, rRNAs and CR are0.028 (from 0.021 in *C*. *capitata* to 0.131 in *B*. *minax*) and −0.194 (from −0.175 in *P*. *utilis* to −0.316 in *B*. *minax*), 0.026 (from 0.019 in *C*. *capitata* to 0.148 in *B*. *minax*) and −0.180 (from −0.170 in *P*. *utilis* to −0.319 in *B*. *minax*), 0.024 (from 0.005 in *P*. *utilis* to 0.055 in *B*. *minax*) and −0.131 (from −0.074 in *B*. *cucurbitae* to −0.182 in *B*. *minax*), 0.048 (minimum) and −0.297 (from −0.263 in *C*. *capitata* to −0.356in *B*. *minax*), −0.045(minimum) and −0.576 (from −0.354 in *D*. *longicornis* to 0.04 in *B*. *cucurbitae*), respectively. The rRNAs and CR of *N*.*asiatica* shows the most marked AT skews compared with the other tephritid mitogenomes, which are significant parallels with the feature in *C*. *capitata* and *C*. *fasciventris*. The CR of *N*. *asiatica*, *C*. *capitata* and *C*. *fasciventris* all show negative AT skews, while that of the other tephritid mitogenomes show positive AT skews (Supplementary Table S2).

Seven PCGs in all Tephritidae species have the same start codons (ATG in *ATP6*, *COII*, *CYTB*, *ND4* and *ND4L*, ATT in *ND2*, TCG in *COI*), and five PCGs (*ATP6*, *ATP8*, *COIII*, *ND4L* and *ND6*) have the same stop TAA codons (Table 3). In *ND5*, the TAT stop codon of *N*. *asiatica* is different from all the other Tephritidae species with TAA or T stop codon.

**Table 3:** Usage of start and stop codons in mitochondrial genome of Tephritidae.

Species	ATP6		ATP8		COI		COII		COIII		CYTB		ND1	
	start	stop	start	stop	start	stop	start	stop	start	stop	start	stop	start	stop
*N*. *asiatica* (*Becker*)	ATG	TAA	ATT	TAA	TCG	TAA	ATG	TAA	ATG	TAA	ATG	TAG	ATA	T
*A*. *fraterculus*	ATG	TAA	ATT	TAA	TCG	TAA	ATG	TAA	ATG	TAA	ATG	TAG	ACA	TAA
*B*. *arecae*	ATG	TAA	GTG	TAA	TCG	TA	ATG	TAA	ATG	TAA	ATG	TAA	ATA	T
*B*. *carambolae*	ATG	TAA	GTG	TAA	TCG	TA	ATG	TAA	ATG	TAA	ATG	T	ATA	T
*B*. *correcta*	ATG	TAA	GTG	TAA	TCG	TA	ATG	TAA	ATG	TAA	ATG	TAG	ATA	T
*B*. *depressa*	ATG	TAA	ATT	TAA	TCG	TAA	ATG	TAA	ATG	TAA	ATG	TAA	ATA	TAA
*B*. *dorsalis*	ATG	TAA	GTG	TAA	TCG	TA	ATG	TAA	ATG	TAA	ATG	TAG	ATA	T
*B*. *latifrons*	ATG	TAA	GTG	TAA	TCG	TA	ATG	TAA	ATG	TAA	ATG	TAA	ATA	T
*B*. *melastomatos*	ATG	TAA	GTG	TAA	TCG	TA	ATG	TAA	ATG	TAA	ATG	T	ATA	T
*B*. *tryoni*	ATG	TAA	GTG	TAA	TCG	TA	ATG	TAA	ATG	TAA	ATG	TAG	ATA	T
*B*. *umbrosa*	ATG	TAA	ATG	TAA	TCG	TA	ATG	TAA	ATG	TAA	ATG	T	ATA	T
*B*. *zonata*	ATG	TAA	GTG	TAA	TCG	TA	ATG	TAA	ATG	TAA	ATG	TAG	ATA	T
*B*. *oleae*	ATG	TAA	ATG	TAA	TCG	TA	ATG	TAA	ATG	TAA	ATG	TAG	ATG	T
*B*. *minax*	ATG	TAA	ATT	TAA	TCG	TA	ATG	TAA	ATG	TAA	ATG	TAG	ATA	T
*B*. *caudate*	ATG	TAA	ATT	TAA	TCG	TAA	ATG	TAA	ATG	TAA	ATG	T	ATA	T
*B*. *cucurbitae*	ATG	TAA	ATT	TAA	TCG	TAA	ATG	TAA	ATG	TAA	ATG	T	ATA	T
*B*. *diaphora*	ATG	TAA	ATT	TAA	TCG	TAA	ATG	TAA	ATG	TAA	ATG	TAG	ATA	T
*B*. *scutellata*	ATG	TAA	ATT	TAA	TCG	TAA	ATG	TAA	ATG	TAA	ATG	TAG	ATA	T
*B*. *tau*	ATG	TAA	ATT	TAA	TCG	TAA	ATG	TAA	ATG	TAA	ATG	TAG	ATA	T
*C*. *capitata*	ATG	TAA	ATT	TAA	TCG	TAA	ATG	TAA	ATG	TAA	ATG	T	ATT	T
*C*. *fasciventris*	ATG	TAA	ATT	TAA	TCG	TAA	ATG	TAA	ATG	TAA	ATG	TAG	ATT	TAA
*D*. *longicornis*	ATG	TAA	ATC	TAA	TCG	TAA	ATG	TAA	ATG	TAA	ATG	T	ATG	T
*P*. *utilis*	ATG	TAA	ATT	TAA	TCG	TAA	ATG	T	ATA	TAA	ATG	TAA	ATA	TAG
**Species**	**ND2**		**ND3**		**ND4**		**ND4L**		**ND5**		**ND6**			
	**start**	**stop**	**start**	**stop**	**start**	**stop**	**start**	**stop**	**start**	**stop**	**start**	**stop**		
*N*. *asiatica* (*Becker*)	ATT	TAA	ATA	TAA	ATG	TAA	ATG	TAA	ATT	TAT	ATT	TAA		
*A*. *fraterculus*	ATT	TAG	ATT	TAA	ATG	TAA	ATG	TAA	ATT	TAA	ATT	TAA		
*B*. *arecae*	ATT	TAA	ATT	T	ATG	TAG	ATG	TAA	ATC	T	ATT	TAA		
*B*. *carambolae*	ATT	TAA	ATT	TAG	ATG	TAG	ATG	TAA	ATT	T	ATT	TAA		
*B*. *correcta*	ATT	TAA	ATT	TAG	ATG	TAG	ATG	TAA	ATT	T	ATT	TAA		
*B*. *depressa*	ATT	TAG	ATC	TAG	ATG	TAA	ATG	TAA	ATT	TAA	ATT	TAA		
*B*. *dorsalis*	ATT	TAA	ATT	T	ATG	TAG	ATG	TAA	ATT	T	ATT	TAA		
*B*. *latifrons*	ATT	TAA	ATT	T	ATG	TAG	ATG	TAA	ATT	T	ATT	TAA		
*B*. *melastomatos*	ATT	TAA	ATC	T	ATG	TAG	ATG	TAA	ATT	T	ATT	TAA		
*B*. *tryoni*	ATT	TAA	ATT	T	ATG	TAG	ATG	TAA	ATT	T	ATC	TAA		
*B*. *umbrosa*	ATT	TAA	ATT	T	ATG	TAG	ATG	TAA	ATT	T	ATT	TAA		
*B*. *zonata*	ATT	TAA	ATT	T	ATG	TAG	ATG	TAA	ATT	T	ATT	TAA		
*B*. *oleae*	ATT	TAA	ATC	TAG	ATG	TAA	ATG	TAA	ATT	TAA	ATC	TAA		
*B*. *minax*	ATT	TAG	ATC	T	ATG	TAA	ATG	TAA	ATT	TAA	ATG	TAA		
*B*. *caudata*	ATT	TAA	ATC	TAG	ATG	TAA	ATG	TAA	ATT	T	ATT	TAA		
*B*. *cucurbitae*	ATT	TAA	ATC	TAG	ATG	TAA	ATG	TAA	ATT	T	ATT	TAA		
*B*. *diaphora*	ATT	TAA	ATC	T	ATG	TAA	ATG	TAA	ATT	T	ATT	TAA		
*B*. *scutellata*	ATT	TAA	ATC	TAG	ATG	TAA	ATG	TAA	ATT	T	ATT	TAA		
*B*. *tau*	ATT	TAA	ATC	TAA	ATG	TAA	ATG	TAA	ATT	T	ATT	TAA		
*C*. *capitata*	ATT	TAA	ATA	TAA	ATG	TAA	ATG	TAA	ATT	T	ATT	TAA		
*C*. *fasciventris*	ATT	TAA	ATA	TAA	ATG	TAA	ATG	TAA	ATT	TAA	ATT	TAA		
*D*. *longicornis*	ATT	TAG	ATA	TAG	ATG	TAG	ATG	TAA	ATT	T	ATC	TAA		
*P*. *utilis*	ATT	TAA	ATT	TAA	ATG	TAA	ATG	TAA	ATT	T	ATA	TAA		

Phylogenetic relationship of Tephritid fruit flies based on molecular data has been reported by several researchers and there exist some arguments for a long period.

The relationship between subgenus *Zegodacus* and other subgenus of *Bactrocera* is questionable. White suggested that subgenera *Zeugodacus* should split from *Bactrocera* to combine with *Dacus* genus to form a new genus—*Zeugodacus* from morphological evidence^8^. Latter, a lot of studies support the view from molecular level. Segura *et al*. reported the phylogenetic relationships among 23 tephritid species using the utilizing sequence of *CYTB*, *tRNA* ^*Se*r^ and *ND1* genes. The result indicated *Bactrocera cucurbitae* is close to genus *Dacus* rather than other subgenus of *Bactrocera* ^9^. Krosch *et al*. rebuilt the phylogenetic tree of 125 species based on *16S rRNA*, *COI*, *COII* and *white eye* genes to figure out the Tribe Dacini relationship and similarly the tree showed that *Zeugodacus* is the sister group to *Dacus* not *Bactrocera*. They suggested *Zeugodacus* should raise up to genus level^10^. Virgilio *et al*. also came to the result through the phylogenetic tree using two datasets. Dataset 1 was an alignment of 2,338 bp consisted of *COI*, *16S rRNA*, *tRNA* ^*pro*^, *ND6* and *period* included 98 vouchers and dataset 2 was an alignment of 1,200 bp consisted of *COI* and *16 S rRNA* included 159 vouchers^11^. In this study, we confirmed that subgenera *Zeugodacus* are closer to genus *Dacus* but distinct from other subgenera (*Bactrocera*, *Daculus *and *Tetradacus*) of *Bactrocera* genus from mitochondrial genome data level.

Han and Ro reconstructed the phylogeny of the family Tephritidae by mitochondrial *12 S*, *16 S*, and *COII* gene fragments using 79 tephritid species. Phylogenetic trees suggested that Dacini and Ceratitidini are sister group which both of them belong to Dacinae and have distance to *Anastrepha* which belong to Toxotrypanini^12^. While Krosch *et al*. found *Anastrepha ludens* which belongs to Trypetinae subfamily was closer to Dacini (Dacinae subfamily) than to *C*. *capitata* based on *16S rRNA*, *COI*, *COII* and *white eye* genes^10^. Fernández *et al*. constructed the phylogenetic tree using the neighbour-joining method based on *COII* gene representing six genera (*Ceratitis*, *Rhagoletis*, *Dacus*, *Bactrocera*, *Anastrepha* and *Toxotrypan*) of the family. The result also showed that *Anastrepha* and *Bactrocera* cluster in one branch while *Ceratitis* formed another branch individually^13^. Nakahara and Murajiuse used a 1.3 kb portion of mitochondrial DNA containing the *tRNA* ^*Leu*^ and flanking *COI* and *COII* regions for phylogenetic analyses. The result also shows that Dacini seems more closely related to *Anastrepha* than to the Ceratitidini^14^. Our research also drew the same conclusion that *Anastrepha fraterculus* is closer to Dacini rather than to *C*. *capitata* using the published mitochondrial genome data (5 of 6 datasets posterior probabilities are 1.00 and ML bootstraps are 100 for Bayesian and ML analyses separately) which implicates that we should reconsider the phylogenetic relationships between Dacinae and Trypetinae according to the molecular evidence.

There is also an argument about the phylogenetic status of the genus *Neoceratitis*, most of which are sequenced by four mitochondrial and one nuclear gene fragment (*COI*, *16 S*, *tRNA* ^*Pro*^, *ND6*, *period*). Barr and McPheron investigated phylogenetic relationships within Ceratitidina and showed that *Neoceratitis* might be sister taxa to *Ceratitis* along with *Carpophthoromyia* and *Capparimyia* ^15^. Based on the gene fragments (*COI*, *16S*, *tRNA* ^*Pro*^, *ND6*, *period*), the study of  Virgilio *et al*. strongly supported that the genera *Ceratitis* and *Neoceratitis* were sister taxa using Bayesian approach and maximum likelihood (ML) (Bayesian PP = 1.00, ML bootstrap support = 91)^11^. So far, various studies, all of which expounding with the sample *Neoceratitis cyanescens*, have shown the close relationship between the two genera, *Ceratitis* and *Neoceratitis* ^9^. Based on the previous studies mentioned above, the phylogenetic position between the genera *Ceratitis* and *Neoceratitis* was not well resolved. Thus we expected that the complete mitochondrial genome sequence of *N*. *asiatica* could make some contributions towards the phylogeny reconstruction of subtribe Ceratitidina.

In this study, the Bayesian and ML reconstructions place the two genera *Ceratitis* (*C*. *capitata*) and *Neoceratitis* (*N*. *asiatica*) together, which means they may be sister taxa. Limited to the data of complete mitochondrial genome in different Tephritidae species, exploring the relationship between the two genera *Ceratitis* and *Neoceratitis* still needs more researches.

## Materials and Methods

### Sample collection and DNA extraction

The *N*. *asiatica* samples were collected in Ningxia province, China and preserved in 100% ethanol. They were identified based on morphological characteristics. Genomic DNA was extracted from individual *N*. *asiatica* adult using the DNeasy DNA Extraction kit (QIAGEN).

### Mitogenome sequencing and annotation

Genomic DNA library preparation and sequencing were carried out by Berry Genomics sequencing company (Beijing, China). Genomic DNA was fragmented with Bioruptor to an average insert size of 250 bp and sequenced on Illumina Hiseq 2500. Part of *cox1* gene was sequenced as the “anchor” to reconstruct the mitochondrial genome of *N*. *asiatica* using a general insect primer pairLCO1490/HCO2198^16^. We picked up the mitochondrial genome sequence with “map to reference” strategy and mapped all cleaned NGS reads to the “anchor” by Geneious R10.0^17^. The parameters we set for assembly were: 1) minimum overlap identity 95%, 2) minimum overlap 50 bp, 3) maximum 5% gaps per read, and 4) maximum gap size 20 bp.

Thirteen protein-coding genes and two rRNA genes were identified by BLAST searches in NCBI (http://www.ncbi.nlm.nih.gov/) and then confirmed by alignment with homologous genes from other 22 Tephritid species available in GenBank. The tRNA genes were identified using the tRNAscan-SE^18^ and MITOS WebServer^19^. The circular map of *N*. *asiatica* complete mitochondrial genome was generated and annotated using Geneious. The start/stop codon usages were analysed by DNAMAN 8.0. The composition of skew was calculated manually based on the formula: AT skew = (A − T)/(A + T) and GC skew = (G − C)/(G + C)^20^. The sequin file was edited and submitted to NCBI (NCBI GenBank accession number MF434829).

### Phylogenetic analysis

A total of 25species of Diptera species were used in phylogenetic analysis, including 23Tephritidae and 2 outgroups species from Drosophilidae. Six datasets were used to build phylogenetic trees: 1) PCG123: 13 protein-coding genes (all three codon positions included); 2) PCG123 + rRNA: 13 protein-coding genes and 2 rRNA genes; 3) PCG123 + rRNA + tRNA: 13 protein-coding genes, 2 rRNA genes and 22 tRNA genes; 4) PCG12: 13 protein-coding genes (first two codon positions included) with; 5) PCG12 + rRNA: 13 protein-coding genes and 2 rRNA genes; 6) PCG12 + rRNA + tRNA: 13 protein-coding genes, 2 rRNA genes and 22 tRNA genes.

MrBayes v.3.2.5^21^ and a PHYML^22^ online web server were used to analyze the six datasets under GTR + I + G model. The model was selected using Jmodeltest 2.1.7^23^. In Bayesian analysis, two simultaneous runs of 1,000,000 generations were conducted for the matrix. Each one was sampled every 200 generations with a burn-in of 25%. Trees inferred prior to stationarity were discarded as burn-in, and the remaining were used to construct a 50% majority rule consensus tree. The ML analysis was conducted with 1,000 bootstraps. Phylogenetic trees were viewed and edited by FigTree v.1.4.3^24^. Sequences were aligned using ClustalW with the default parameters implemented in MEGA 5.0^25^. The ambiguous positions in the genes alignment were filtered with Gblocks v0.91b^26^. The aligned sequences of each gene were concatenated using SequenceMatrix v1.7^27^.

## Electronic supplementary material

Supplementary Information
