# Supplementary material for: The mitochondrial genome of the wolfberry fruit fly, Neoceratitis asiatica (Becker) (Diptera: Tephritidae) and the phylogeny of Neoceratitis Hendel genus
Source: Sci Rep. 2017 Nov 30;7:16612. doi: 10.1038/s41598-017-16929-7 (PMC5709374; doi:10.1038/s41598-017-16929-7)
Supplement: Supplementary file 1 — Supplementary Information [file 41598_2017_16929_MOESM1_ESM.docx]

The mitochondrial genome of the wolfberry fruit fly, *Neoceratitis asiatica* (Becker) (Diptera: Tephritidae) and the phylogeny of *Neoceratitis* Hendel genus

Yun Su^1,a^, Yue Zhang^1,a^, Shiqian Feng^1,a^, Jia He^2^, Zihua Zhao^1^, Zhenzhen Bai^1^, Lijun Liu^1*^, Rong Zhang^2,*^, Zhihong Li^1,*^

^1^Department of Entomology, College of Plant Protection, China Agricultural University, Beijing 100193, China

^2^The Institute of Plant Protection, Ningxia Academy of Agriculture and Forestry Sciences, Yinchuan 750002, China

^a^These authors contributed equally.

**Correspondence:**

Zhihong Li; 537 Horticulture Building, China Agricultural University, Haidian District, Beijing 100193, P. R. China; +86 10 62733000; [lizh@cau.edu.cn](mailto:lizh@cau.edu.cn)

Rong Zhang; The Institute of Plant Protection, Ningxia Academy of Agriculture and Forestry Sciences, Xingqing District, Yinchuan 750002, P. R. China; [yczhrnx@163.com](mailto:yczhrnx@163.com)

Lijun Liu; 537 Horticulture Building, China Agricultural University, Haidian District, Beijing 100193, P. R. China; +86 10 62733000; [ljliu@cau.edu.cn](mailto:ljliu@cau.edu.cn)

**Table S1. GenBank accession** **information for mitochondrial genome of Tephritidae and genes for phylogenetics (by early June, 2017).**

| tribe | Genus | Subgenus | Species | Accession Number | Population |
| --- | --- | --- | --- | --- | --- |
| Toxotrypanini | [*Anastrepha*](https://www.ncbi.nlm.nih.gov/Taxonomy/Browser/wwwtax.cgi?mode=Undef&id=28585&lvl=3&keep=1&srchmode=1&unlock) |  | *A. fraterculus* | KX926433 | Ibagué, Tolima, Colombia |
| Dacini | *Bactrocera* | *Bactrocera* | *B. arecae* | KR233259 | Kuala Lumpur, Malaysia |
|  |  |  | *B. carambolae* | EF014414 | Japan |
|  |  |  | *B. correcta* | JX456552 | Yunnan, China |
|  |  |  | *B. dorsalis* | DQ845759 | Guangdong, China |
|  |  |  |  | DQ917577 | Guangdong, China |
|  |  |  |  | DQ917578 | Malaysia |
|  |  |  |  | DQ995281 | Philippines |
|  |  |  |  | KX534207 | Kenya |
|  |  |  | *B. latifrons* | KT881556 | Malaysia |
|  |  |  | *B. melastomatos* | KT881557 | Malaysia |
|  |  |  | *B. tryoni* | HQ130030 | / |
|  |  |  | *B. umbrosa* | KT881558 | Malaysia |
|  |  |  | *B. zonata* | KP296150 | Ranchi, India |
|  |  | *Daculus* | *B. oleae* | AY210702 | Mirandela, Portugal |
|  |  |  |  | AY210703 | Siena, Italy |
|  |  |  |  | GU108459 | Italy |
|  |  |  |  | GU108460 | Israel |
|  |  |  |  | GU108461 | Turkey |
|  |  |  |  | GU108462 | South Africa |
|  |  |  |  | GU108463 | Pakistan |
|  |  |  |  | GU108464 | Italy |
|  |  |  |  | GU108465 | Turkey |
|  |  |  |  | GU108466 | Kenya |
|  |  |  |  | GU108467 | Pakistan |
|  |  |  |  | GU108468 | Algeria |
|  |  |  |  | GU108469 | Pakistan |
|  |  |  |  | GU108470 | Italy |
|  |  |  |  | GU108471 | Italy |
|  |  |  |  | GU108472 | Israel |
|  |  |  |  | GU108473 | Portugal |
|  |  |  |  | GU108474 | Morocco |
|  |  |  |  | GU108475 | USA |
|  |  |  |  | GU108476 | Kenya |
|  |  |  |  | GU108477 | Pakistan |
|  |  |  |  | GU108478 | South Africa |
|  |  |  |  | GU108479 | USA |
|  |  |  |  | KR677101 | Iran |
|  |  |  |  | KR677102 | Iran |
|  |  |  |  | KR677103 | Iran |
|  |  |  |  | KR677104 | Iran |
|  |  |  |  | KR677105 | Iran |
|  |  |  |  | KR677106 | Iran |
|  |  |  |  | KR677107 | Iran |
|  |  | *Tetradacus* | *B. minax* | HM776033 | Chongqing, China |
|  |  | *Zeugodacus* | *B. caudate* | KT625491 | Malaysia |
|  |  |  |  | KT625492 | Indonesia |
|  |  |  | *B. cucurbitae* | JN635562 | Yunnan, China |
|  |  |  | *B. depressa* | KY131831 | South Korea |
|  |  |  | *B. diaphora* | KT159730 | Chongqing, China |
|  |  |  | *B. scutellata* | KP722192 | Guangdong, China |
|  |  |  |  | KT159731 | / |
|  |  |  | *B. tau* | KP711431 | Guangdong, China |
| Cecidocharini | *Procecidochares* |  | *P. utilis* | KC355248 | Yunnan, China |
| Ceratitidini | *Ceratitis* | *Ceratitis* | *C. capitata* | AJ242872 | Laboratory Strain |
|  |  |  | *C.* *fasciventris* | KY436396 | Seibersdorf, Austria |
|  | *Neoceratitis* |  | *N. asiatica* | MF434829 | Ningxia, China |
| Dacini | *Dacus* | *Callantra* | *D. longicornis* | KX345846 | Yunnan, China |

The grey part represents the mitochondrial genome data used in the phylogenetic analysis.

**Table S2.** **Nucleotide composition information for the mitochondrial genome of Tephritidae.**

| **Species** | **GenBank Number** | **Whole mtDNA** | | | | **PCGs** | | | | **tRNAs** | | | | **rRNAs** | | | | **CR** | | | |
| --- | --- | --- | --- | --- | --- | --- | --- | --- | --- | --- | --- | --- | --- | --- | --- | --- | --- | --- | --- | --- | --- |
|  |  | **length** | **(A+T) %** | **AT skew** | **GC skew** | **length** | **(A+T) %** | **AT skew** | **GC skew** | **length** | **(A+T) %** | **AT skew** | **GC skew** | **length** | **(A+T) %** | **AT skew** | **GC skew** | **length** | **(A+T) %** | **AT skew** | **GC skew** |
| *A. fraterculus* | KX926433 | 16739 | 77.1 | 0.064 | -0.205 | 11194 | 74.5 | 0.066 | -0.197 | 1479 | 76.9 | 0.03 | -0.126 | 2116 | 80.1 | 0.076 | -0.266 | 1182 | 87.5 | 0.05 | -0.27 |
| *B. arecae* | KR233259 | 15900 | 72.26 | 0.081 | -0.259 | 11182 | 69.66 | 0.086 | -0.265 | 1467 | 74.47 | 0.035 | -0.131 | 2111 | 77.12 | 0.095 | -0.329 | 952 | 86.03 | 0.06 | -0.128 |
| *B. carambolae* | EF014414 | 15915 | 73.55 | 0.066 | -0.224 | 11192 | 71.1 | 0.072 | -0.228 | 1466 | 75.1 | 0.019 | -0.096 | 2113 | 77.57 | 0.079 | -0.304 | 950 | 87.89 | 0.049 | -0.13 |
| *B. correcta* | JX456552 | 15936 | 73.17 | 0.063 | -0.222 | 11192 | 71.22 | 0.07 | -0.221 | 1470 | 75.31 | 0.024 | -0.113 | 2117 | 77.85 | 0.08 | -0.322 | 949 | 78.61 | 0.019 | -0.163 |
| *B. dorsalis* | DQ845759 | 15915 | 73.58 | 0.068 | -0.228 | 11185 | 71.12 | 0.073 | -0.235 | 1467 | 75.19 | 0.017 | -0.093 | 2123 | 77.81 | 0.076 | -0.287 | 949 | 88.09 | 0.062 | -0.186 |
|  | DQ917577 | 15917 | 73.6 | 0.065 | -0.227 | 11190 | 71.1 | 0.072 | -0.229 | 1466 | 75.1 | 0.02 | -0.097 | 2114 | 77.7 | 0.076 | -0.282 | 951 | 88.1 | 0.058 | -0.153 |
|  | DQ917578 | 15915 | 73.52 | 0.066 | -0.226 | 11190 | 71.04 | 0.073 | -0.234 | 1466 | 75.38 | 0.024 | -0.086 | 2114 | 77.72 | 0.069 | -0.287 | 950 | 88.21 | 0.064 | -0.161 |
|  | DQ995281 | 15915 | 73.63 | 0.066 | -0.224 | 11192 | 71.18 | 0.071 | -0.229 | 1466 | 75.31 | 0.02 | -0.099 | 2114 | 77.67 | 0.074 | -0.292 | 949 | 88.2 | 0.061 | -0.179 |
|  | KX534207 | 15911 | 73.6 | 0.068 | -0.227 | 11191 | 71.1 | 0.075 | -0.236 | 1466 | 75.4 | 0.016 | -0.073 | 2119 | 77.8 | 0.075 | -0.288 | 949 | 88.1 | 0.062 | -0.193 |
| *B. latifrons* | KT881556 | 15977 | 71.11 | 0.088 | -0.266 | 11184 | 68.1 | 0.099 | -0.275 | 1466 | 73.47 | 0.042 | -0.136 | 2120 | 77.22 | 0.084 | -0.317 | 953 | 86.78 | 0.069 | -0.238 |
| *B. melastomatos* | KT881557 | 15954 | 73.79 | 0.073 | -0.251 | 11187 | 71.23 | 0.078 | -0.254 | 1468 | 75.48 | 0.029 | -0.133 | 2114 | 78.1 | 0.087 | -0.305 | 953 | 88.98 | 0.059 | -0.257 |
| *B. tryoni* | HQ130030 | 15925 | 72.42 | 0.07 | -0.227 | 11187 | 69.61 | 0.074 | -0.236 | 1467 | 74.98 | 0.022 | -0.09 | 2115 | 77.73 | 0.073 | -0.295 | 951 | 86.96 | 0.083 | -0.129 |
| *B. umbrosa* | KT881558 | 15898 | 70.49 | 0.084 | -0.244 | 11187 | 67.27 | 0.09 | -0.245 | 1465 | 74.13 | 0.042 | -0.145 | 2120 | 77.03 | 0.094 | -0.343 | 944 | 86.23 | 0.049 | -0.046 |
| *B. zonata* | KP296150 | 15935 | 73.34 | 0.065 | -0.223 | 11190 | 70.99 | 0.07 | -0.224 | 1469 | 74.95 | 0.023 | -0.103 | 2120 | 78.07 | 0.077 | -0.308 | 950 | 84.42 | 0.04 | -0.216 |
| *B. oleae* | AY210702 | 15815 | 72.63 | 0.088 | -0.28 | 11189 | 70.18 | 0.091 | -0.288 | 1466 | 74.76 | 0.04 | -0.13 | 2116 | 77.13 | 0.099 | -0.347 | 949 | 86.93 | 0.091 | -0.177 |
|  | AY210703 | 15815 | 72.59 | 0.088 | -0.28 | 11189 | 70.15 | 0.091 | -0.287 | 1466 | 74.69 | 0.041 | -0.132 | 2116 | 77.08 | 0.099 | -0.344 | 949 | 86.83 | 0.09 | -0.184 |
|  | GU108459 | 15814 | 72.6 | 0.088 | -0.281 | 11184 | 70.18 | 0.092 | -0.289 | 1466 | 74.76 | 0.04 | -0.13 | 2116 | 77.08 | 0.099 | -0.344 | 948 | 86.71 | 0.083 | -0.19 |
|  | GU108460 | 15817 | 72.63 | 0.089 | -0.282 | 11184 | 70.21 | 0.092 | -0.291 | 1466 | 74.69 | 0.039 | -0.127 | 2116 | 77.08 | 0.1 | -0.348 | 951 | 86.86 | 0.087 | -0.184 |
|  | GU108461 | 15816 | 72.65 | 0.089 | -0.282 | 11184 | 70.22 | 0.092 | -0.291 | 1466 | 74.76 | 0.04 | -0.13 | 2116 | 77.08 | 0.1 | -0.344 | 950 | 86.95 | 0.09 | -0.194 |
|  | GU108462 | 15814 | 72.63 | 0.089 | -0.281 | 11184 | 70.23 | 0.091 | -0.29 | 1466 | 74.69 | 0.039 | -0.127 | 2115 | 77.07 | 0.101 | -0.348 | 949 | 86.72 | 0.089 | -0.19 |
|  | GU108463 | 15821 | 72.52 | 0.089 | -0.281 | 11184 | 70.07 | 0.093 | -0.29 | 1466 | 74.56 | 0.038 | -0.126 | 2116 | 76.89 | 0.1 | -0.35 | 949 | 87.04 | 0.085 | -0.187 |
|  | GU108464 | 15814 | 72.59 | 0.089 | -0.281 | 11184 | 70.16 | 0.091 | -0.29 | 1466 | 74.76 | 0.04 | -0.13 | 2116 | 77.08 | 0.099 | -0.344 | 948 | 86.71 | 0.088 | -0.19 |
|  | GU108465 | 15816 | 72.65 | 0.088 | -0.28 | 11184 | 70.23 | 0.091 | -0.287 | 1467 | 74.64 | 0.039 | -0.124 | 2115 | 77.07 | 0.099 | -0.348 | 950 | 86.95 | 0.087 | -0.194 |
|  | GU108466 | 15813 | 72.63 | 0.089 | -0.281 | 11184 | 70.23 | 0.091 | -0.29 | 1466 | 74.69 | 0.039 | -0.127 | 2115 | 77.07 | 0.101 | -0.348 | 948 | 86.71 | 0.09 | -0.19 |
|  | GU108467 | 15821 | 72.52 | 0.089 | -0.281 | 11184 | 70.07 | 0.093 | -0.29 | 1466 | 74.56 | 0.038 | -0.126 | 2116 | 76.89 | 0.1 | -0.35 | 949 | 87.04 | 0.085 | -0.187 |
|  | GU108468 | 15815 | 72.61 | 0.088 | -0.279 | 11184 | 70.19 | 0.091 | -0.289 | 1466 | 74.69 | 0.039 | -0.127 | 2116 | 77.08 | 0.099 | -0.344 | 949 | 86.83 | 0.09 | -0.184 |
|  | GU108469 | 15815 | 72.53 | 0.089 | -0.281 | 11184 | 70.09 | 0.093 | -0.29 | 1467 | 74.64 | 0.039 | -0.124 | 2115 | 76.83 | 0.1 | -0.351 | 949 | 87.04 | 0.085 | -0.187 |
|  | GU108470 | 15815 | 72.61 | 0.089 | -0.281 | 11184 | 70.19 | 0.091 | -0.29 | 1466 | 74.76 | 0.04 | -0.13 | 2116 | 77.08 | 0.099 | -0.344 | 949 | 86.72 | 0.089 | -0.19 |
|  | GU108471 | 15813 | 72.6 | 0.089 | -0.28 | 11184 | 70.16 | 0.092 | -0.289 | 1466 | 74.76 | 0.04 | -0.13 | 2116 | 77.08 | 0.099 | -0.344 | 947 | 86.8 | 0.088 | -0.184 |
|  | GU108472 | 15814 | 72.66 | 0.088 | -0.28 | 11184 | 70.25 | 0.091 | -0.289 | 1467 | 74.64 | 0.039 | -0.124 | 2115 | 77.07 | 0.099 | -0.348 | 948 | 86.92 | 0.085 | -0.194 |
|  | GU108473 | 15815 | 72.61 | 0.089 | -0.28 | 11184 | 70.2 | 0.091 | -0.289 | 1466 | 74.69 | 0.039 | -0.127 | 2116 | 77.08 | 0.099 | -0.344 | 949 | 86.72 | 0.094 | -0.175 |
|  | GU108474 | 15817 | 72.61 | 0.089 | -0.279 | 11184 | 70.15 | 0.091 | -0.288 | 1466 | 74.76 | 0.04 | -0.13 | 2116 | 77.08 | 0.1 | -0.348 | 951 | 86.96 | 0.091 | -0.177 |
|  | GU108475 | 15819 | 72.67 | 0.089 | -0.283 | 11184 | 70.23 | 0.093 | -0.293 | 1466 | 74.69 | 0.039 | -0.127 | 2116 | 77.13 | 0.099 | -0.347 | 949 | 86.93 | 0.088 | -0.177 |
|  | GU108476 | 15816 | 72.62 | 0.088 | -0.279 | 11184 | 70.22 | 0.091 | -0.287 | 1466 | 74.69 | 0.039 | -0.127 | 2116 | 76.98 | 0.101 | -0.347 | 950 | 86.84 | 0.091 | -0.2 |
|  | GU108477 | 15815 | 72.52 | 0.089 | -0.281 | 11184 | 70.07 | 0.093 | -0.29 | 1466 | 74.62 | 0.038 | -0.124 | 2115 | 76.83 | 0.1 | -0.351 | 950 | 87.16 | 0.087 | -0.197 |
|  | GU108478 | 15815 | 72.64 | 0.089 | -0.282 | 11184 | 70.23 | 0.092 | -0.29 | 1466 | 74.69 | 0.039 | -0.127 | 2115 | 77.07 | 0.101 | -0.348 | 950 | 86.74 | 0.09 | -0.19 |
|  | GU108479 | 15820 | 72.67 | 0.09 | -0.283 | 11184 | 70.23 | 0.093 | -0.293 | 1466 | 74.69 | 0.039 | -0.127 | 2116 | 77.13 | 0.099 | -0.347 | 950 | 86.95 | 0.09 | -0.177 |
|  | KR677101 | 15816 | 72.6 | 0.088 | -0.277 | 11190 | 70.2 | 0.091 | -0.289 | 1466 | 74.8 | 0.04 | -0.13 | 2116 | 77.1 | 0.097 | -0.345 | 950 | 86.8 | 0.088 | -0.188 |
|  | KR677102 | 15816 | 72.6 | 0.088 | -0.282 | 11190 | 70.2 | 0.091 | -0.289 | 1466 | 74.8 | 0.04 | -0.13 | 2116 | 77.1 | 0.097 | -0.345 | 950 | 86.8 | 0.088 | -0.188 |
|  | KR677103 | 15815 | 72.6 | 0.088 | -0.28 | 11190 | 70.2 | 0.091 | -0.289 | 1400 | 74.8 | 0.043 | -0.143 | 2116 | 77.1 | 0.1 | -0.345 | 949 | 86.7 | 0.089 | -0.188 |
|  | KR677104 | 15816 | 72.6 | 0.088 | -0.28 | 11190 | 70.2 | 0.091 | -0.289 | 1466 | 74.6 | 0.038 | -0.126 | 2116 | 77.1 | 0.1 | -0.345 | 950 | 86.8 | 0.088 | -0.188 |
|  | KR677105 | 15816 | 72.6 | 0.088 | -0.28 | 11190 | 70.2 | 0.091 | -0.289 | 1466 | 74.7 | 0.039 | -0.126 | 2116 | 77.1 | 0.1 | -0.345 | 950 | 86.8 | 0.088 | -0.188 |
|  | KR677106 | 15815 | 72.6 | 0.088 | -0.282 | 11190 | 70.2 | 0.091 | -0.289 | 1466 | 74.8 | 0.04 | -0.135 | 2115 | 77 | 0.099 | -0.348 | 950 | 86.9 | 0.086 | -0.197 |
|  | KR677107 | 15816 | 72.6 | 0.088 | -0.277 | 11190 | 70.2 | 0.091 | -0.289 | 1466 | 74.8 | 0.04 | -0.126 | 2116 | 77.1 | 0.1 | -0.345 | 950 | 86.8 | 0.088 | -0.188 |
| *B. minax* | HM776033 | 16043 | 67.28 | 0.131 | -0.316 | 11183 | 64.3 | 0.148 | -0.319 | 1466 | 72.31 | 0.055 | -0.182 | 2115 | 73.71 | 0.121 | -0.356 | 1141 | 77.65 | 0.081 | -0.333 |
| *B. caudate* | KT625491 | 15866 | 73.15 | 0.074 | -0.252 | 11193 | 71.01 | 0.083 | -0.26 | 1472 | 74.6 | 0.018 | -0.102 | 2122 | 77.8 | 0.075 | -0.291 | 944 | 83.69 | 0.063 | -0.26 |
|  | KT625492 | 15885 | 72.84 | 0.076 | -0.255 | 11193 | 70.39 | 0.087 | -0.262 | 1473 | 74.75 | 0.015 | -0.102 | 2122 | 77.71 | 0.075 | -0.29 | 941 | 85.02 | 0.06 | -0.291 |
| *B. cucurbitae* | JN635562 | 15825 | 72.89 | 0.047 | -0.213 | 11190 | 70.71 | 0.049 | -0.226 | 1467 | 75.12 | 0.005 | -0.074 | 2110 | 77.82 | 0.08 | -0.303 | 946 | 82.35 | 0.014 | 0.042 |
| *B. depressa* | KY131831 | 15832 | 71.3 | 0.094 | -0.273 | 11187 | 68.8 | 0.102 | -0.276 | 1469 | 74.4 | 0.04 | -0.137 | 2060 | 76.8 | 0.073 | -0.31 | 1004 | 81.8 | 0.142 | -0.311 |
| *B. diaphora* | KT159730 | 15890 | 74.1 | 0.065 | -0.241 | 11186 | 72.11 | 0.071 | -0.243 | 1471 | 74.71 | 0.021 | -0.113 | 2120 | 77.83 | 0.076 | -0.306 | 946 | 85.1 | 0.061 | -0.305 |
| *B. scutellata* | KP722192 | 15915 | 72.95 | 0.074 | -0.257 | 11273 | 70.73 | 0.081 | -0.262 | 1468 | 74.39 | 0.016 | -0.106 | 2124 | 77.82 | 0.079 | -0.316 | 1011 | 84.87 | 0.072 | -0.262 |
|  | KT159731 | 15850 | 73 | 0.077 | -0.259 | 11270 | 70.7 | 0.83 | -0.267 | 1468 | 74.5 | 0.023 | -0.109 | 2122 | 77.7 | 0.081 | -0.318 | 945 | 84.7 | 0.072 | -0.268 |
| *B. tau* | KP711431 | 15687 | 73.28 | 0.06 | -0.221 | 11273 | 71.4 | 0.059 | -0.225 | 1471 | 74.92 | 0.015 | -0.084 | 2119 | 77.73 | 0.073 | -0.301 | 801 | 83.75 | 0.119 | -0.132 |
| *C. capitata* | AJ242872 | 15980 | 77.48 | 0.021 | -0.185 | 11272 | 75.59 | 0.019 | -0.178 | 1472 | 76.77 | 0.021 | -0.094 | 2123 | 80.22 | 0.058 | -0.267 | 1004 | 91.14 | -0.01 | -0.258 |
| *C. fasciventris* | KY436396 | 16017 | 77.2 | 0.023 | -0.193 | 11263 | 75.2 | 0.023 | -0.185 | 1471 | 76.8 | 0.016 | -0.095 | 2129 | 80.1 | 0.054 | -0.286 | 1024 | 90.2 | -0.024 | -0.122 |
| *D. longicornis* | KX345846 | 16253 | 72.33 | 0.101 | -0.293 | 11208 | 69.4 | 0.105 | -0.301 | 1481 | 74.81 | 0.052 | -0.126 | 2129 | 77.17 | 0.087 | -0.33 | 1343 | 85.26 | 0.146 | -0.354 |
| *N. asiatica* | MF434829 | 15481 | 79 | 0.028 | -0.194 | 11194 | 77.9 | 0.026 | -0.18 | 1485 | 77.9 | 0.024 | -0.131 | 2149 | 81.5 | 0.048 | -0.297 | 397 | 88.2 | -0.045 | -0.576 |
| *P. utilis* | KC355248 | 15922 | 80.83 | 0.037 | -0.175 | 11193 | 78.9 | 0.041 | -0.17 | 1501 | 80.61 | 0.005 | -0.1 | 2236 | 85.69 | 0.051 | -0.3 | 928 | 90.52 | 0.015 | -0.045 |
